# Mouse and Human Genetic Analyses Associate Kalirin with Ventral Striatal Activation during Impulsivity and with Alcohol Misuse

**DOI:** 10.3389/fgene.2016.00052

**Published:** 2016-04-07

**Authors:** Yolanda Peña-Oliver, Fabiana M. Carvalho, Sandra Sanchez-Roige, Erin B. Quinlan, Tianye Jia, Tom Walker-Tilley, Stuart L. Rulten, Frances M. G. Pearl, Tobias Banaschewski, Gareth J. Barker, Arun L. W. Bokde, Christian Büchel, Patricia J. Conrod, Herta Flor, Jürgen Gallinat, Hugh Garavan, Andreas Heinz, Penny Gowland, Marie-Laure Paillere Martinot, Tomáš Paus, Marcella Rietschel, Trevor W. Robbins, Michael N. Smolka, Gunter Schumann, David N. Stephens, for the IMAGEN Consortium

**Affiliations:** ^1^School of Psychology, University of SussexBrighton, UK; ^2^Department of Psychology, University of CambridgeCambridge, UK; ^3^Institute of Psychiatry, Psychology and Neurosciences, Kings CollegeLondon, UK; ^4^MRC Social, Genetic and Developmental Psychiatry CentreLondon, UK; ^5^Genome Damage and Stability Centre, University of SussexBrighton, UK; ^6^School of Life Sciences, University of SussexBrighton, UK; ^7^Central Institute of Mental Health, Medical Faculty Mannheim/Heidelberg UniversityMannheim, Germany; ^8^Institute of Neuroscience, Trinity College DublinDublin, Ireland; ^9^Universitätsklinikum Hamburg EppendorfHamburg, Germany; ^10^Departments of Psychiatry and Psychology, University of VermontBurlington, VT, USA; ^11^Department of Child and Adolescent Psychiatry, Psychosomatics, and Psychotherapy, Charité-UniversitätsmedizinBerlin, Germany; ^12^School of Psychology, University of NottinghamNottingham, UK; ^13^INSERM, UMR 1000, Research Unit Imaging and Psychiatry, IFR49, CEA, DSV, I2BM-Service Hospitalier Frédéric JoliotOrsay, France; ^14^Rotman Research Institute, University of TorontoToronto, ON, Canada; ^15^Department of Psychology and Psychiatry, University of TorontoToronto, ON, Canada; ^16^Department of Psychiatry and Psychotherapy, Technische Universität DresdenDresden, Germany

**Keywords:** impulsivity, binge drinking, adolescent, fMRI, BXD recombinant inbred strains, monetary incentive delay, 5-choice serial reaction time task

## Abstract

Impulsivity is associated with a spectrum of psychiatric disorders including drug addiction. To investigate genetic associations with impulsivity and initiation of drug taking, we took a two-step approach. First, we identified genes whose expression level in prefrontal cortex, striatum and accumbens were associated with impulsive behavior in the 5-choice serial reaction time task across 10 BXD recombinant inbred (BXD RI) mouse strains and their progenitor C57BL/6J and DBA2/J strains. Behavioral data were correlated with regional gene expression using *GeneNetwork* (www.genenetwork.org), to identify 44 genes whose probability of association with impulsivity exceeded a false discovery rate of < 0.05. We then interrogated the IMAGEN database of 1423 adolescents for potential associations of SNPs in human homologs of those genes identified in the mouse study, with brain activation during impulsive performance in the Monetary Incentive Delay task, and with novelty seeking scores from the Temperament and Character Inventory, as well as alcohol experience. There was a significant overall association between the human homologs of impulsivity-related genes and percentage of premature responses in the MID task and with fMRI BOLD-response in ventral striatum (VS) during reward anticipation. In contrast, no significant association was found between the polygenic scores and anterior cingulate cortex activation. Univariate association analyses revealed that the G allele (major) of the intronic SNP rs6438839 in the KALRN gene was significantly associated with increased VS activation. Additionally, the A-allele (minor) of KALRN intronic SNP rs4634050, belonging to the same haplotype block, was associated with increased frequency of binge drinking.

## Introduction

The ability to inhibit inappropriate or undesirable responses plays a fundamental role in successful human behavior. Impulsivity, the failure to control such unplanned and inappropriate actions, is widely implicated in the development and maintenance of addictive behavior (Verdejo-García et al., 2008; Dick et al., 2010), both as increased tendency to engage in risky choices, and as impaired control over drug and alcohol use once initiated. Although most human studies relating impulsivity to addictive behaviors (Verdejo-García et al., 2008) have been based upon self-report questionnaires such as the Barratt Impulsivity Scale (BIS; Patton et al., 1995), these questionnaires do not distinguish separate aspects of impulsivity (Robbins et al., 2012), which have also been extensively characterized using animal models (Evenden, 1999). However, recent advances in neuropsychological testing, which include neuroimaging as well as psychometric characterizations, allow more objective and precise assessments of those separate aspects of impulsivity. Such assessments suggest distinct behavioral endophenotypes for impulsive behaviors (Robbins et al., 2012) and provide the opportunity for integration of data obtained from putatively homologous measures in animal and human subjects (Sanchez-Roige et al., 2014a).

Although drug and alcohol abuse may themselves lead to impulsive behavior as a result of drug-induced changes in fronto-striatal systems (Duka et al., 2011), it is also clear that genetic factors contribute to addiction, possibly by predisposing to initiation of drug taking (Schneider et al., 2012; Whelan et al., 2012). Here we ask whether genes that are associated with poor behavioral control contribute to adolescent alcohol use and abuse. We first used a mouse genetic approach to identify candidate genes associated with one form of impulsivity, a reduced ability to wait to respond to obtain a reward, resulting in loss of the reward. We then examined potential associations of these candidate genes with a related measure of impulsivity, as well as with risk taking and alcohol use in human adolescence, a time when limited experience of drug or alcohol abuse was unlikely to have contributed to the development of loss of control by impairing fronto-striatal function. An association was also examined between the candidate genes and brain activation during reward anticipation, specifically in the ventral striatum (VS) and anterior cingulate cortex (ACC) considering their role in impulsivity and anticipatory responding (Muir et al., 1996; McClure et al., 2004; Hariri et al., 2006; Beck et al., 2009). We thus hoped to identify potential genetic factors that contribute to experimentation with, and abuse of, alcohol at an early stage.

## Materials and methods

In our mouse study we used a panel of 10 BXD recombinant inbred strains, plus their progenitor C57BL/6J (B6) and DBA2/J (D2) strains, to compare performance in measures of “waiting” impulsivity in the 5-choice serial reaction time task (5-CSRTT; Robbins, 2002; Robinson et al., 2009; Sanchez-Roige et al., 2012). We then correlated performance in these measures with gene expression data from prefrontal cortex, nucleus accumbens, and full striatum from Affymetrix microarrays [*GeneNetwork* database (www.genenetwork.org)] to identify genes potentially associated with the behavioral phenotypes (Wang et al., 2003).

In a second step, we used the candidate genes identified in the mouse model as having a high association with impulsive behavior (*p* < 3.13 × 10^−5^) to interrogate a GWAS database obtained from healthy human adolescents for gene variants (SNPs) that were associated with a related measure of impulsivity. All data from IMAGEN project are available from a dedicated database: https://imagen.cea.fr.

Although human versions of the 5-CSRTT have recently been developed (Sanchez-Roige et al., 2014a; Voon et al., 2014), these were not available at the time of our study. Instead, we used an analogous measure derived from the Monetary Incentive Delay task (Balodis and Potenza, 2015). The MID assesses how quickly a subject responds to a target presented on a video display to obtain a reward, whose value is signaled at the start of each trial. Speed of response is influenced by knowledge of the anticipated reward value (Wrase et al., 2007). The target location differs across reward magnitudes, but is fixed for any particular magnitude. In order to derive a measure analogous to the mouse 5-CSRTT (Dalley et al., 2011), we considered only responses made on the screen following information on reward size, but before the target appeared (i.e., in anticipation of target presentation; premature responses), and their associated brain activation. As human 5-CSRTT impulsivity is heightened in individuals with a family history of alcoholism (Sanchez-Roige et al., under review), and associated with binge drinking (Sanchez-Roige et al., 2014a), we also considered the relationship of these measures to alcohol abuse. By limiting our analysis to those genes identified in the mouse, we were able to increase the power of our statistical analysis of the human database.

## Mouse studies

### Subjects

Nine to fifteen male mice from C57BL/6J (B6), DBA/2J (D2) and 10 BXD recombinant inbred strains (BXD 5, 11, 12, 18, 21, 29, 31, 32, 33, and 36) were purchased from The Jackson Laboratory (Bar Harbor, Maine, USA) and were imported at 5–8 weeks of age. Mice were accustomed to the University of Sussex facility for 1 month before testing in the 5-CSRTT. Strain BXD11 was aggressive, and needed to be housed individually. In order to maintain similar conditions across groups, all other strains were also singly-housed. Animals were maintained on a 12 h light/dark cycle (lights off at 7 p.m.), at a temperature of 19–21°C and 50% humidity. Before starting 5-CSRTT training, mice were food restricted to reduce their body weights to 85% of their free-feeding weight. Water was available *ad libitum*. All experiments were approved by the institutional ethics committee and were performed under United Kingdom legislation on animal experimentation [Animal (Scientific Procedures) Act, 1986].

### Five-choice serial reaction time task (5-CSRTT)

Testing of performance followed the protocol previously described (Peña-Oliver et al., 2012). Briefly, mice were trained in eight mouse operant chambers (Med Associates Inc., St. Albans, Vermont, USA). The left wall of the chamber contained five apertures fitted with infrared detectors to detect nose-poke responses. The apertures were illuminated by a yellow stimulus light located inside each aperture, mice being required to nose-poke into a briefly illuminated aperture to obtain reinforcers (correct responses). The right wall of the chamber contained a receptacle in which the liquid reinforcer (0.01 ml of 30% condensed milk solution) was delivered. Head entries into the food magazine were recorded by an infrared photo-cell beam. The presentation of stimuli and the recording of the responses were controlled by MED-PC for Windows (Med Associates Inc., St. Albans, Vermont, USA). Failures to respond (omissions) were punished by extinguishing all lights for 5 s, as were entries into the wrong aperture (incorrect responses). Following stable performance in the last stage of training [Stimulus duration = 1.8 s; Limited hold (maximum duration to make a response after stimulus presentation) = 5 s; inter-trial interval (ITI) = 5 s; TO = 5 s; >75% accuracy, < 25% omissions for 2 consecutive days], mice were presented with a long ITI session (ITI = 10 vs. 5 s during baseline), which promotes the emergence of responses prior to cue-onset (premature responses; Dalley et al., 2007). The main outcome included in the analysis was premature responding (premature responses/[correct responses + incorrect responses + omissions + premature responses] × 100; see Oliver et al., 2009; Walker et al., 2011 for further details of the 5-CSRTT protocol).

## Data analysis

### Statistical analysis

Statistical analyses were performed using the “Statistical Package for Social Sciences” (SPSS, version 18.0) for the variable “Percentage of premature responding”: premature responses/(correct responses + incorrect responses + omissions + premature responses) × 100.

One-way analysis of variance (ANOVA) was performed, following arcsine transformation [x′ = 2 arcsine (√(x/100))] of percentage of premature responding, for baseline and the long ITI session to elucidate differences between strains, followed by Tukey's *post-hoc* tests.

### Calculation of heritability

Narrow sense heritability (h^2^) of percentage of premature responding was calculated as the *R*^2^-value from the one-way ANOVA as described by Belknap (1998). Thus, Heritability (h^2^) = SSM (between-subjects sum of squares)/SST (total sum of squares). This estimate reflects the proportion of total phenotypic variation that is due to additive genetic factors, excluding genetic dominance effects, epistatic interactions, or strain-unique environmental influences.

### Identification of candidate genes through the BXD approach

The variable representing impulsivity (percent premature responses in the long ITI session) was entered into the Gene Network database (record ID/16606) and was correlated with microarray-based gene expression data available within www.GeneNetwork.org with prefrontal cortex [Gene Network Codes: VCU BXD PFC Sal M430 2.0(Dec06) RMA, accession number GN135], nucleus accumbens [Gene Network Codes: VCU BXD NA Sal M430 2.0 (Oct07) RMA, accession number GN156], and striatum [Gene Network Codes: HBP Rosen Striatum M430V2 (Apr05) PDNN Clean, accession number GN68], brain areas which have been demonstrated to play a role in impulsivity and inhibitory control (Robbins, 2002; Christakou et al., 2004; Dalley et al., 2011). Spearman rank-order correlations were carried out using the tool available within www.GeneNetwork.org.

We excluded those genes that fell below the criterion determined by a false discovery rate (FDR) of 0.05, following the procedure explained in Benjamini et al. (2001).

## Human studies

### Participants

One thousand four hundred and twenty three adolescents assessed as part of the IMAGEN project (Schumann et al., 2010) were included in the association analyses of neuroimaging data [mean age = 14.43 years, standard deviation (SD) = 0.41 years; 48.7% male; 87.4% right-handed; verbal IQ = 111.46, SD = 15.24; performance IQ = 108.06, SD = 14.32]. Thirty-nine cases were excluded in both polygenic score and the association analyses of impulsivity and binge drinking due to incomplete behavioral data sets. Local ethics research committees at each site approved the study.

### Genotyping

DNA purification and genotyping was performed by the Centre National de Génotypage (Paris, France). DNA was extracted from whole blood samples (~10 ml) preserved in BD Vacutainer EDTA tubes (Becton, Dickinson and Company, Oxford, UK) using Gentra Puregene Blood Kit (QIAGEN Inc., Valencia, CA, USA) according to the manufacturer's instructions. Genotype information was collected at 582,982 markers, including 5000 base-pair extensions for both 3 and 5′ ends of each gene, to capture both 3 and 5′ untranslated regions, using the Illumina HumanHap610 Genotyping BeadChip (Illumina, San Diego, CA, USA). SNPs with call rates of < 98%, minor allele frequency < 1% or deviation from the Hardy-Weinberg equilibrium (*p* ≤ 1 × 10^−4^) were excluded from the analyses. Individuals with an ambiguous sex code, excessive missing genotypes (failure rate >2%), and outlying heterozygosity (heterozygosity rate of 3 SDs from the mean) were also excluded. Identity-by-state similarity was used to estimate cryptic relatedness for each pair of individuals using PLINK software. Closely related individuals with identity-by-descent (IBD > 0.1875) were eliminated from the subsequent analysis. Population stratification for the genotyping data was examined by principal component analysis (PCA) using EIGENSTRAT software. The four HapMap populations were used as reference groups in the PCA analysis and individuals with divergent ancestry (from CEU) were also excluded.

### Polygenetic score analysis

One thousand eight hundred and ten SNPs in 37 human homologs of mouse candidate genes (Table 1) were used for polygenetic score analysis performed in PLINK. After pruning for strong linkage disequilibrium (pairwise *r*^2^ threshold of 0.5 and 50 SNP sliding window), 830 SNPs remained. For each phenotype of interest, 10 random re-samplings were made to partition participants into 70% discovery and 30% target samples. We carried out the following steps for each of these 10 pairs of samples: (1) we calculated associations between each SNP and phenotype in the discovery sample using linear regression with study site, handedness and gender as covariates; (2) we selected alleles based on a widely used moderate significance threshold *p* < 0.5 (Dudbridge, 2013) to arrive at a set of “testing SNPs” for each phenotype in the discovery sample; (3) in the target sample we calculated the score of each “testing SNP” for each phenotype for each individual as the number of alleles multiplied by the corresponding regression coefficient from the discovery sample, these scores were then summed up across all the “testing SNPs” to give the final polygenetic score; (4) we calculated the semi-partial correlations between polygenetic scores (i.e., predicted phenotypes) and observed phenotypes in the target sample, where only the latter was controlled for study site, handedness and gender on observed phenotype because the polygenetic scores are expected to be uncorrelated to these covariates. This process yields 10 semi-partial correlations and *p*-values for each phenotype, corresponding to the 10 random re-samplings of participants. An exact *p*-value was then calculated from each set of 10 *p*-values to give an overall interpretation of the polygenetic score analysis.

**Table 1:** **List of genes whose expression in different mouse brain areas was significantly correlated with percentage of premature responses in the long ITI session of the 5-CSRTT using a false discovery rate of 0.05**.

**Record ID**	**Gene symbol**	Gene symbol (HUMAN)^*^	**Description**	**CHR**	**No. of SNPs tested**	**rho**	***p***
**PREFRONTAL CORTEX**							
1419593_at	Greb1	GREB1	Growth retardation in Dh male mice, QTL 1	2	30	0.96	1.69E-08
1458740_at	4933411D12Rik	No homolog	RIKEN cDNA 4933411D12 gene	–	–	−0.95	1.04E-07
1422141_s_at	Csprs	No homolog	Component of Sp100-rs	–	–	0.95	4.31E-07
1442473_at	KIAA1217	KIAA1217	BC02665 protein	10	239	−0.95	4.31E-07
1451555_at	Nln	NLN	Neurolysin (metallopeptidase M3 family)	5	37	−0.95	4.31E-07
1445329_at	Pycr1	PYCR1	ESTs	17	0	−0.94	1.36E-06
1459780_at	Trp53	TP53	Transformation related protein 53	17	6	−0.94	1.36E-06
1422970_at	Mxd3	MXD3	Max dimerization protein 3	5	3	−0.93	3.58E-06
1437418_at	Ern1	ERN1	Endoplasmic reticulum (ER) to nucleus signaling 1	17	13	0.93	3.58E-06
1446514_at	Dpp10	DPP10	Dipeptidylpeptidase 10	2	271	−0.93	3.58E-06
1458832_at	Ghr	GHR	Growth hormone receptor	5	32	−0.93	3.58E-06
1458924_at	Sumo1	SUMO1	Small ubiquitin-like modifier 1 (SMT3 suppressor of mif two 3 homolog 1)	2	2	−0.93	3.58E-06
1459793_s_at	Ghiso	LYRM5	RIKEN cDNA 4930469P12	12	11	−0.93	3.58E-06
1429987_at	9930013L23Rik	KIAA1199	RIKEN cDNA 9930013L23 gene	15	60	0.92	8.15E-06
1436521_at	Slc36a2	SLC36A2	Solute carrier family 36 (proton/amino acid symporter), member 2	5	11	0.92	8.15E-06
1432386_a_at	Phf7	PHF7	PHD finger protein 7	3	2	−0.92	8.15E-06
1439660_at	E030045D18Rik	HIVEP3	RIKEN cDNA E030045D18 gene	1	107	−0.92	8.15E-06
1444140_at	Pum1	PUM1	Pumilio 1 (PUF family RNA-binding protein)	1	11	−0.92	8.15E-06
1457184_at	4930488L10Rik	FRMD6	RIKEN cDNA 4930488L10 gene	14	95	−0.92	8.15E-06
1459214_at	Odz2	TENM2	Odd Oz/ten-m homolog 2	5	221	−0.92	8.15E-06
1423619_at	Rasd1	RASD1	RAS, dexamethasone-induced 1	17	5	0.91	1.67E-05
1427555_at	BC032281	MLL2	cDNA sequence BC032281	12	5	0.91	1.67E-05
1452471_at	Il17rd	IL17RD	Interleukin 17 receptor D	3	23	0.91	1.67E-05
1426306_a_at	Maged2	MAGED2	Melanoma antigen, family D, 2	X	–	−0.91	1.67E-05
1429410_at	Eny2	ENY2	e(y)2 protein	8	2	−0.91	1.67E-05
1437076_at	A930017M01Rik	No homolog	RIKEN cDNA A930017M01 gene	–	–	−0.91	1.67E-05
1440022_at	Poldip3	POLDIP3	Polymerase (DNA-directed), delta interacting protein 3	22	10	−0.91	1.67E-05
1441795_at	Snapc4	SNAPC4	Small nuclear RNA activating complex, polypeptide 4	9	8	−0.91	1.67E-05
1450100_a_at	Tcerg1	TCERG1	Transcription elongation regulator 1 (CA150)	5	11	−0.91	1.67E-05
1458045_at	Odz4	TENM4	Odd Oz/ten-m homolog 4 (Drosophila)	11	261	−0.91	1.67E-05
1420667_at	Doc2b	DOC2B	Double C2, beta	–	–	0.90	3.13E-05
1435697_a_at	Pscdbp	CYTIP	Pleckstrin homology, Sec7 and coiled-coil domains, binding protein	2	11	0.90	3.13E-05
1439551_at	BC006965	No homolog	16 days embryo head cDNA, RIKEN full-length enriched library, clone:C130043L22 product:unknown EST, full insert sequence.	–	–	0.90	3.13E-05
1418909_at	Ermap	ERMAP	Erythroblast membrane-associated protein	1	10	−0.90	3.13E-05
1427379_at	2610204M12Rik	ATFZIP	Mus musculus, clone IMAGE:3987110, mRNA, partial cds	12	16	−0.90	3.13E-05
1429834_a_at	C11orf2	VPS51	Protein fat-free homolog (human chromosome 11 open reading frame 2)	11	9	−0.90	3.13E-05
1432944_at	Golsyn	SUBU	Golgi-localized syntaphilin-related protein (activity-dependent presynaptic assembly, kinesin motor-adaptor complex)	8	31	−0.90	3.13E-05
1439652_at	Selenbp2	SELEMBP1	Selenium binding protein 2	1	9	−0.90	3.13E-05
1442554_s_at	Kalrn	KALRN	Kalirin, RhoGEF kinase (huntingtin-associated protein interacting protein duo)	3	156	−0.90	3.13E-05
1453512_at	Mbnl2	MBNL2	Muscleblind-like 2	13	48	−0.90	3.13E-05
1456138_at	E130115E03Rik	LYPD6	RIKEN cDNA E130115E03 gene	2	24	−0.90	3.13E-05
1460575_at	D3Ertd194e	EIF2A	DNA segment, Chr 3, ERATO Doi 194, expressed	3	12	−0.90	3.13E-05
**NUCLEUS ACCUMBENS**							
1458842_at	Odz1	TENM1	Odd Oz/ten-m 1 (Rhs family protein)	X	–	−0.95	4.31E-07
**STRIATUM**							
1435297_at	Gja9	GJA9	Gap junction membrane channel protein alpha 9 (rod pathway critical connexin)	1	8	−0.94	1.04E-06

*The table shows the results for percentage of premature responding (impulsivity) for PFC, nucleus accumbens and striatal gene expression. Also included are the names of the human homologs, their chromosomal location, and the number of SNPs identified and used in the current analysis. CHR: chromosome, rho: value of the Spearman's rank correlation coefficient, p: significance value of the correlation*.

* *Gene symbol according to UCSC hg18 NCBI36/Version Mar 2006*.

### The exact test for the re-sampling results

The exact *p*-values of polygenetic score analyses with m random re-samplings can be calculated based on two levels of information:

First, the semi-partial correlation between the predicted and observed phenotypes is expected to be positive if the alternative hypothesis is true, i.e., the SNPs tested do contribute to the phenotype of interest. In other words the more positive the correlation observed, the more likely is the null hypothesis to be rejected. Under the null hypothesis, we can then calculate the probability of observing *n* or more positive correlations from m random re-samplings as:

(1) $$\begin{array}{l} \frac{\underset{k=n}{\overset{m}{\sum }}\left(\begin{array}{c} m\\ k \end{array}\right)}{2^{m}}; \end{array}$$

Second, when the positive correlation was observed, the corresponding *p*-value should be uniformly distributed between 0 and 0.5 under the null hypothesis. This means the more closely those *p*-values approach 0, the more likely the null hypothesis is to be rejected. In such a circumstance, we can denote max(*p*) as the maximum observed *p*-value (one-sided) among these positive correlations, and then calculate the chance of observing *n* independently uniformly distributed variables within [0, 0.5] which are all smaller than max(*p*) as

$$\begin{array}{l} {\left(\frac{\max\left(p\right)}{0.5}\right)}^{n}. \end{array}$$

By joining the above two levels of information, we can then have the exact *p*-value of *m* resampling as:

$$\begin{array}{l} {\frac{\underset{k=n+1}{\overset{m}{\sum }}\left(\begin{array}{c} m\\ k \end{array}\right)+\left(\begin{array}{c} m\\ n \end{array}\right)\left(\frac{\max\left(p\right)}{0.5}\right)}{2^{m}}}^{n}, \end{array}$$

where if *n* = *m*, the first term of numerator is 0. In our case, *m* = 10.

### Enrichment analysis

The enrichment analysis was conducted to compare if a group of *P*-values were indeed different from a random counterpart. Such a process was widely adopted in analyses related to verify performance of candidate genes (Subramanian et al., 2005). The number of SNP *P*-values below a given threshold was counted as the test statistic. Permutation process was adopted to generate a null distribution of the test statistic from random selected genes, which keep the same LD structure of genes and therefore are comparable to the original candidate genes. The percentage of null distribution which is larger than the test statistic from original candidate genes is then the *P*-value of enrichment analysis. To avoid over fitting, we performed enrichment analysis with SNP *P*-value threshold at both 0.05 and 0.10.

### MRI acquisition

Full details of the entire MRI acquisition protocols and quality checks are described elsewhere (Schumann et al., 2010), including an extensive period of standardization across MRI scanners. The effect of MRI site, gender, and handedness were controlled by adding them as nuisance covariates in all statistical analyses.

### Functional MRI paradigm and data analysis: The monetary incentive delay task

Participants performed the MID task during whole-brain 3-T blood oxygen level functional magnetic resonance imaging. This version of the MID task used in the present study included 66 10-s trials. During each trial, participants were presented with one of three cue shapes (cue, 250 ms) on the left or right side of the screen. The cue indicated where a target (white square) would appear after a variable interval (4000–4500 ms) and whether 0, 2, or 10 points could be won in that particular trial. Participants were instructed to respond with a left or right button press as soon as the target appeared. After the response, subsequent feedback notified the participants how many points were won in the trial, as well as their cumulative total points (outcome, 1450 ms). A tracking algorithm adjusted task difficulty based on target duration (between 100 and 300 ms) such that each participant successfully responded on ~66% of trials. Each five points were converted in one small chocolate candy.

Functional MRI data were analyzed with SPM8 (Statistical Parametric Mapping, http://www.fil.ion.ucl.ac.uk/spm). Slice-time correction was conducted to adjust for time differences due to multi-slice imaging acquisition, all volumes were aligned to the first volume and non-linear warping was performed to an EPI template. Images were then smoothed with a Gaussian kernel of 5 mm full-width at half-maximum. At the first level of analysis, changes in the BOLD response for each subject were assessed by linear combinations at the individual subject level. For each experimental condition, each trial (i.e., reward anticipation high gain; reward feedback) was convolved with the hemodynamic response function to form regressors that account for potential noise variance associated with the processing of reward anticipation. Estimated movement parameters were added to the design matrix in the form of 18 additional columns (3 translations, 3 rotations, 3 quadratic, and 3 cubic translations, and each 3 translations with a shift of ±1 TR). Single-subject contrast images were normalized to Montreal Neurological Institute (MNI) space. The normalized and smoothed single-subject contrast images were then taken to a second-level random effects analysis. Gender, handedness, and data center location were included as covariates in all analyses. The ventral striatum (VS) and anterior cingulate gyrus (ACC) regions-of-interest (ROI) were created and extracted from the whole-brain analysis map yielded by “anticipation big win vs. baseline” contrast using the Marsbar toolbox (http://marsbar.sourceforge.net). Only successful “hit” trials were included and no behavioral or genetic data were introduced as a covariant in this analysis. The ROIs were delineated on the basis of previous literature reports: VS ROI was based on O'Doherty et al. (2004) (xyz = ±15, −9, 9, radius of 9 mm), and the ACC ROI was based on Noonan et al. (2011) (xyz = ±9, 21, 37, radius of 8 mm). The ROIs combined activation of the left and right hemisphere. In addition to the ROI analysis, we also carried out an exploratory whole-brain analysis in which the SNP rs6438839 was included as a covariant. However, it revealed no significant effect at a whole brain level of the SNP rs6438839 at the threshold family-wise error (FWE) *p* < 0.05.

### Behavioral characterization

The Temperament and Character Inventory-Revised (TCI-R) (Cloninger et al., 1991) was used to obtain a novelty seeking score of the participants. Binge drinking phenotype was characterized using an adapted version of the 2007 ESPAD questionnaire (Hibell et al., 2009). In the current study it was defined based on the frequency of binge drinking over the last month (consumption of 5 or more drinks in a row). The variable is coded in a 6-point scale ranging from 0 (“never”) to 5 (“10 times or more”). From the 1384 adolescents included in the binge drinking analysis, 133 reported binge drinking as defined by a score of 1–5. We limited our questionnaire to the prior 30 days to ensure accuracy, as reports of more distant events are less reliable. Nevertheless, subsequent analysis indicates that data for lifetime binge drinking (344 reported) were similar. The behavioral data acquired during the scanning session were used to calculate the percentage of premature responses for “big win” trials: (number of responses given before the visual target was present/total number of responses) × 100. This variable was used as comparable phenotype of impulsivity to the 5-CSRTT “percentage of premature responding” variable.

## Results

### Strain differences in impulsivity in the 5-CSRTT

**Figure 1:**
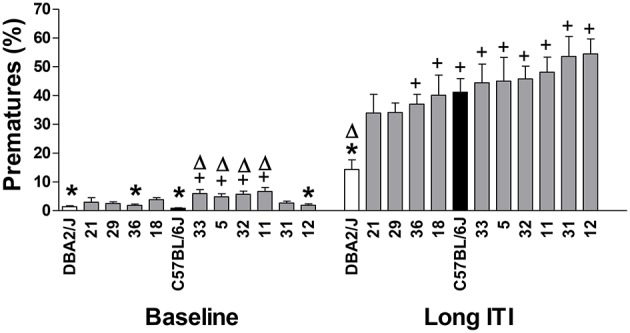
**Performance of C57BL/6J (C57, black bars), DBA/2J (DBA, clear bars) and the 10 BXD recombinant inbred strains (gray bars) in the 5-CSRTT for the baseline (mean of the last three sessions on stage 6) and the long ITI session**. The values represent the mean ± SE Δ*p* < 0.05, significantly different from C57, ^+^ *p* < 0.05 significantly different from DBA, ^*^p < 0.05 vs. the strain with the highest value in the same session.

Figure 1 shows the percentage of premature responses during baseline and the long ITI sessions. Strain means are arranged in ascending order for the values of the long ITI session for the variable % premature responses. Significant strain differences appeared both under baseline (training) conditions [*F*_(11, 94)_ = 6.88; *p* = 2.112E-08] and during a challenge in which the ITI was increased from the training value of 5-s to one of 10-s [*F*_(11, 94)_ = 6.39; *p* = 5.121E-08]. Although no differences in impulsivity were found between B6 and D2 mice during baseline conditions, upon the introduction of the long ITI sessions, in which all strains increased premature responding, we found B6 mice to display higher levels of premature responding than D2 mice, which showed the lowest value of all the strains tested; B6 mice showed intermediate values of impulsivity in comparison with other strains (see Figure 1). Heritability estimates for impulsivity during baseline and the long ITI session gave genetic effect sizes of 44 and 42% respectively, indicating that under these conditions, for both phases more than 40% of the variance was attributable to strain. However, comparison of strain data obtained from the training phase (baseline) and following the introduction of the long ITI showed only a weak correlation between strain values for premature responses (Rho = 0.373; n.s., data not shown).

### Associations between impulsivity in the mouse and gene expression in genenetwork

We correlated our impulsive phenotypic variable from the long ITI session with gene expression data available in the *GeneNetwork* database (www.GeneNetwork.org). Table 1 shows the list of genes whose expression levels in prefrontal cortex (PFC), nucleus accumbens and striatum correlated at a level better than the false discovery rate (FDR) of 0.05 with the variable percentage of premature responding. PFC was the region that showed the greatest number of genes highly correlated with levels of impulsivity (42 out of 44 genes).

### Associations between genes differentially expressed in mouse brain and impulsivity phenotypes in humans

Since a high correlation was found between the mouse gene expression levels and the impulsivity phenotype defined by 5-CSRTT, we investigated whether human homologs of the same genes were also associated with impulsivity phenotypes, or drug taking in adolescent humans. We identified 37 human homologs that were characterized by 1810 SNPs (Table 1).

**Figure 2:**
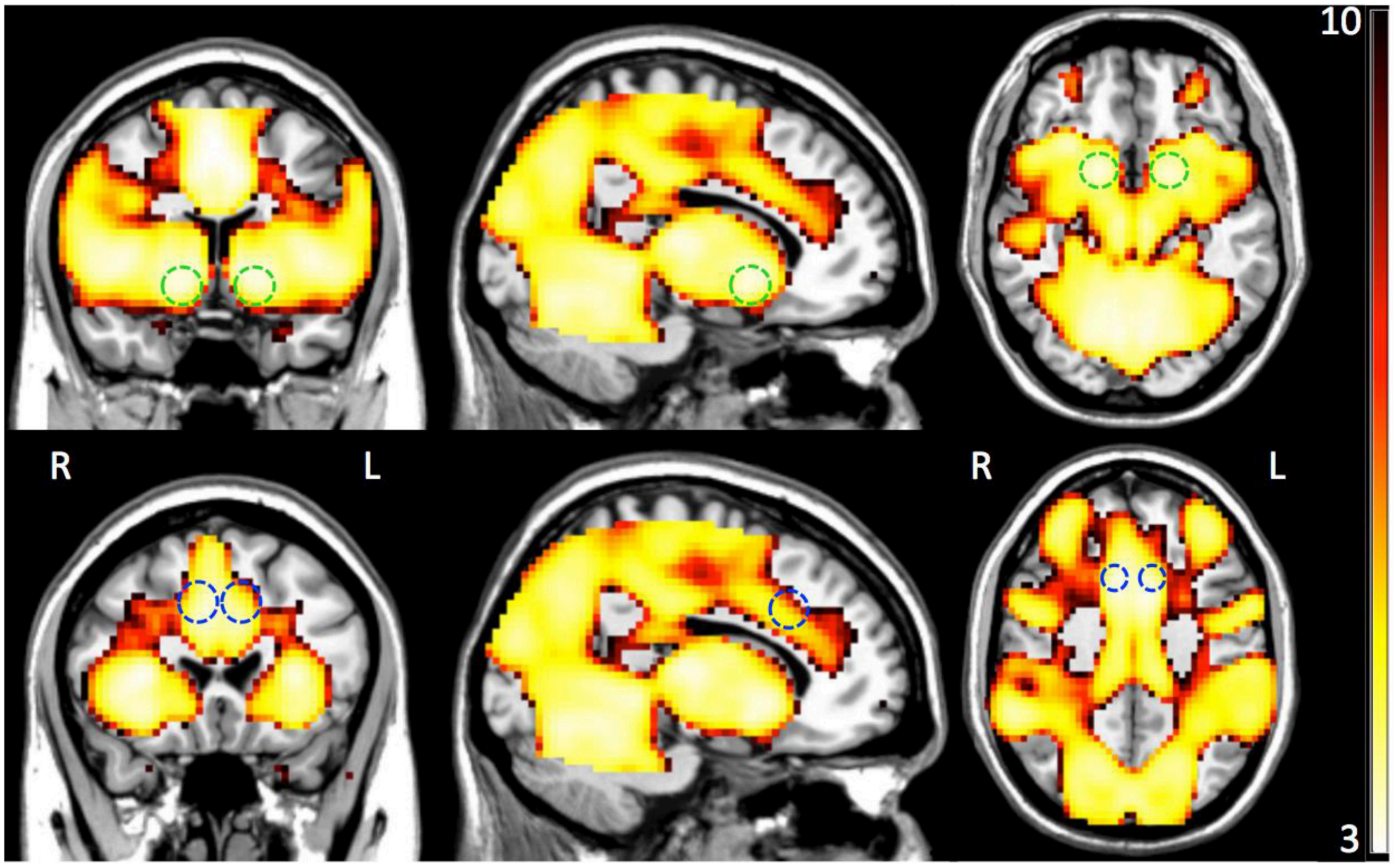
**Group whole-brain positive BOLD map (*n* = 1423) for the contrast Anticipation big win vs. Baseline (FWE *P* < 0.05) with overlay of the two combined ROIs—ventral striatum (VS; top panel) and anterior cingulate cortex (ACC; bottom panel)**. The color bar indicates resulting statistical map *z*-scores.

First, we tested whether the differentially expressed genes as a group were associated with premature responses, as well as ventral striatal activation during the MID task. To this end, we conducted a semi-partial correlation analysis between the polygenic scores (generated in PLINK—see Section Materials and Methods) of all differentially expressed genes and the measure of interest. Premature responses in the MID task occurred in 12.1 ± 10.0 percent of “big win” trials, with a significant association between the polygenic scores and percentage of premature responses (*p* = 6.6 × 10^−3^, Bonferroni corrected). Secondly, we carried out a similar analysis for fMRI BOLD-response in both VS and ACC during reward anticipation. Figure 2 illustrates the whole brain positive BOLD response given by the contrast “Anticipation big win vs. Baseline” (FWE *p* < 0.05) with the two regions-of-interest overlaid (VS: top panel; ACC: bottom panel). There was a significant association between the polygenic scores and the BOLD response for VS during reward anticipation (*p* = 5.9 × 10^−3^, Bonferroni corrected; Table 2), as well as between the polygenic scores and percent premature responding (*p* < 0.001). However, no significant association was found between the polygenic scores and ACC activation. We also found an association with the novelty seeking scale of the TCI-R (*p* = 1.8 × 10^−2^, Bonferroni corrected; Table 2), which has been shown to be associated with impulsiveness (Hur and Bouchard, 1997).

**Table 2:** ***p*-values in the polygenetic score analysis obtained from the 10 target groups for the phenotypes of interest**.

**Phenotype**	**Number of positive semipartial correlations out of 10 target groups**	***p*-value (Bonferroni)**
Novelty Seeking	9	1.8 × 10^−2^
Premature responses	10	6.6 × 10^−3^
left VS	10	5.9 × 10^−3^
right VS	10	6.3 × 10^−3^
left ACC	6	1.0
right ACC	2	1.0

*VS, ventral striatum; ACC, anterior cingulate cortex*.

**Figure 3:**
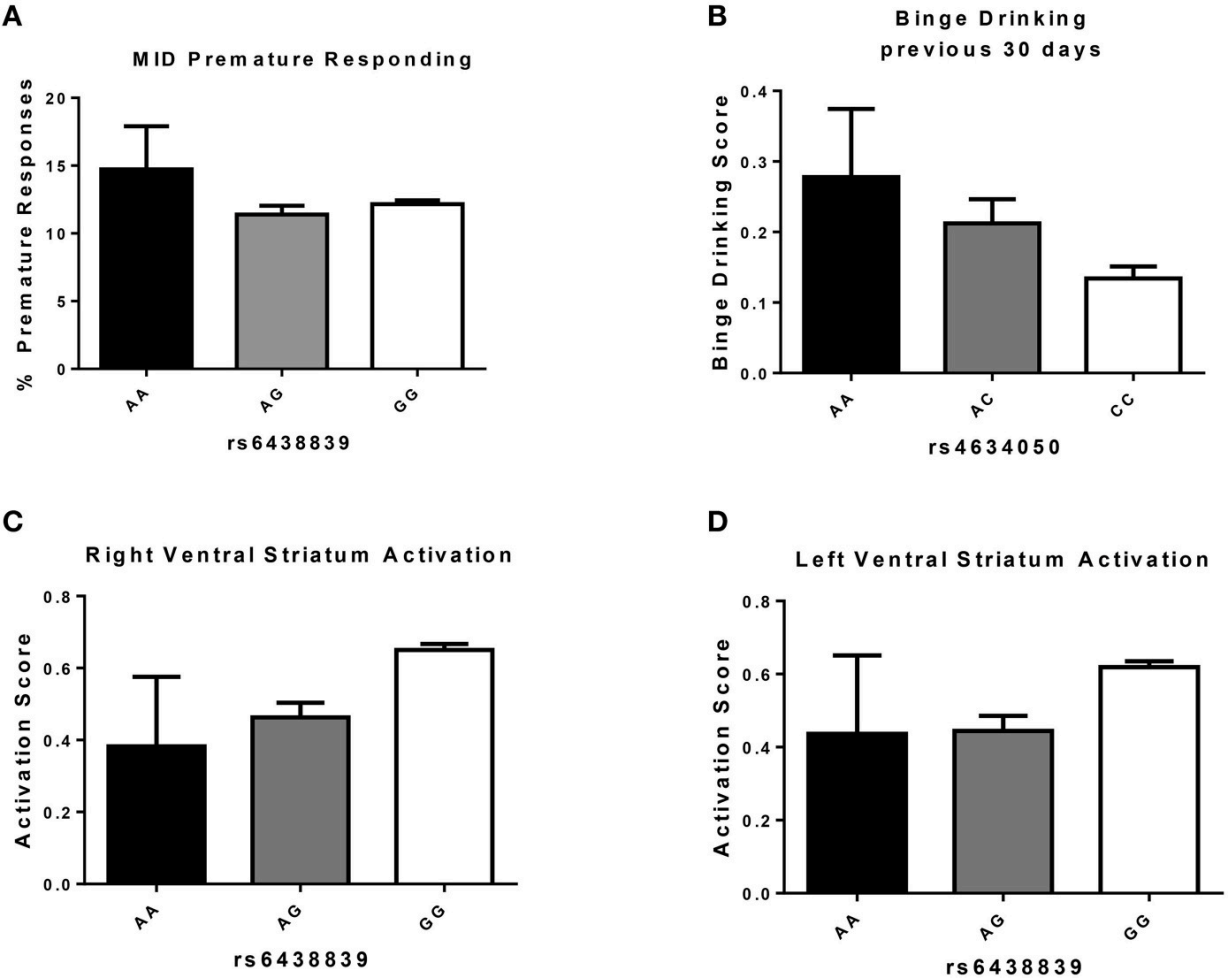
**Measures (Mean ± S.E.) associated with KALRN SNP variations**. **(A)** Percentage of premature responses during “big win” trials in individuals carrying A or G alleles of KALRN SNP rs6438839 (n: AA = 9, AG = 204, GG = 1180); **(B)** Binge Drinking Score [coded in a 6-point scale ranging from 0 (“never”) to 5 (“10 times or more”)] in individuals carrying A or C alleles of KALRN SNP rs634050 (n: AA = 55, AC = 412, CC = 956); **(C)** Activation of Right Ventral Striatum during anticipation of a “big win” in individuals carrying A or G alleles of KALRN SNP rs6438839 (n: AA = 10, AG = 208, GG = 1205); **(D)** Activation of Left Ventral Striatum during anticipation of a “big win” in individuals carrying A or G alleles of KALRN SNP rs6438839 (n: AA = 10, AG = 208, GG = 1205).

In order to identify which SNP(s) were contributing the most to the polygenic association, we carried out univariate association analyses between the same candidate genes/SNPs and phenotypes, using an additive model. Figure 3 shows that the G allele (minor) of the intronic SNP rs6438839 in the *KALRN* gene was significantly associated with increased right VS activation (*p* = 2.14 × 10^−2^, Bonferroni corrected; Table 3), and close to significance for the left VS activation (*p* = 9.97 × 10^−2^ Bonferroni corrected). Nevertheless, the association of this allele with fewer premature responses (Figure 3A) was not significant (*p* = 0.434). Investigation of a possible association between the candidate genes/SNPs with drinking behavior revealed that the A-allele (major) of the intronic SNP rs4634050 in the KALRN gene was significantly associated with an increased frequency of binge drinking (*p* = 4.87 × 10^−2^ Bonferroni corrected; Figure 3B, Table 3). Although only a few SNPs were found significant after Bonferroni correction, the enrichment analysis (Subramanian et al., 2005) between right VS activation and all 1810 SNPs shows that the 37 candidate genes are indeed superior to randomly selected 37 genes with *P* = 0.024 (if comparing SNPs with *P* < 0.05) and *P* = 0.020 (if comparing SNPs with *P* < 0.10), where 500 permutations were applied to simulate random selection as well as maintaining the LD structure within these genes.

**Table 3:** **Significant *p*-values from the univariate association analyses for the KALRN gene**.

**Gene Symbol (CHR)**	**SNP**	**Polymorphism**	**Minor/major allele**	**Phenotype**	***p*-value (Bonferroni)**
KALRN (3)	rs4634050	Intronic	A/C	Binge drinking	4.87E-02
	rs6438839	Intronic	A/G	Right VS	2.14E-02

*VS, ventral striatum*.

Partial correlation analyses (controlling for site, gender, and handedness) were conducted to understand the relationship between the outcome variables that were evaluated against the polygenic score—i.e., binge drinking, premature responding, novelty seeking, and VS/ACC brain activation during the MID task. Among the behavioral variables, the only significant relationship was found between novelty seeking and binge drinking (*r* = 0.156, *p* = 9.5 × 10^−9^, Bonferroni corrected). Ventral striatum activation was significantly associated with premature responding (*r* = 0.124, *p* = 5.0 × 10^−6^ Bonferroni corrected) while ACC was less strongly associated with premature responding (*r* = 0.084, *p* = 0.002 Bonferroni corrected). Ventral striatum and ACC activation were highly correlated with each other (*r* = 0.541, *p* = 6.2 × 10^−103^).

**Figure 4:**
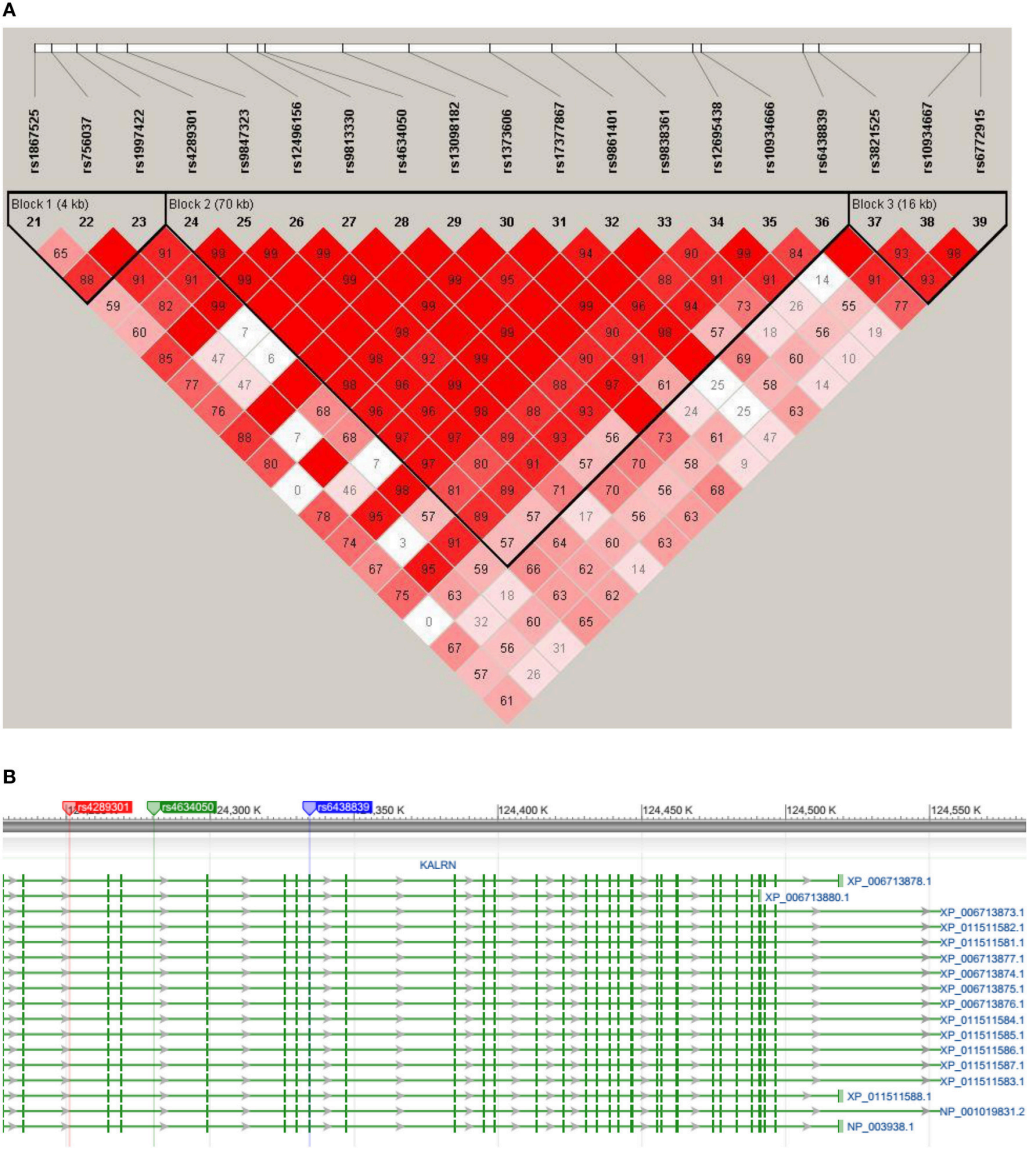
**(A)** The LD plots of KALRN gene surrounding the SNPs of interest. The haplotype blocks are established through the four gamete test where the 4th gamete frequency is set to be higher than 0.05. SNPs of interest, i.e., rs4634050 and rs6438839, are contained in the same haplotype block. **(B)** The illustration of SNP locations on KALRN gene. The flanking SNPs of haplotype block defined in panel **(A)**, i.e., rs4289301 and rs6438839, were highlighted in red and blue, respectively, where the later one is also one of the two target SNPs. The other target SNP, i.e., rs4634050, was also highlighted in green.

An LD structure of KALRN gene was then established. SNPs rs6438839 and rs4634050 belong to the same haplotype block based on the “four gamete test” where the 4th gamete has frequency higher than 0.05 (Figure 4). The haplotype block was then detected to be associated with both right VS activation (*p* = 4.48E-03) and binge drinking behavior (*p* = 4.05 × 10^−3^) in omnibus tests. A further haplotype analysis shows it is Hap8 with frequency 0.0795 that is associated with both phenotypes (*p* = 5.23 × 10^−3^ with right VS activation and *p* = 1.66 × 10^−3^ with binge drinking behavior; Table 4).

**Table 4:** **The results of haplotype analyses for KALRN gene**.

**Haplotype**	**rs4289301**	**rs9847323**	**rs12496156**	**rs9813330**	**rs4634050**	**rs13098182**	**rs1373606**	**rs17377867**	**rs9861401**	**rs9838361**	**rs12695438**	**rs10934666**	**rs6438839**	**Frequency**	**Binge drinking**	**R_VS**
Hap1	C	C	G	G	C	G	C	G	G	G	C	C	G	0.255	9.68E-01	1.94E-02
Hap2	T	T	G	G	C	G	A	G	G	G	T	C	G	0.0173	4.88E-01	3.47E-01
Hap3	T	T	A	G	C	G	A	G	G	T	T	T	G	0.33	5.19E-02	6.00E-01
Hap4	T	T	G	T	A	G	C	G	A	G	C	C	G	0.0873	7.99E-03	2.63E-01
Hap5	T	T	A	G	C	G	A	G	G	T	C	C	G	0.0178	2.49E-01	6.80E-01
Hap6	T	T	G	G	C	G	A	G	G	G	T	C	A	0.0496	4.70E-01	5.79E-03
Hap7	C	C	G	G	C	G	C	A	G	G	C	C	G	0.0846	6.60E-01	6.04E-02
Hap8	T	T	G	T	A	A	C	G	A	G	C	C	G	0.0795	1.66E-03	5.23E-03
														Omnibus	4.05E-03	4.48E-03

*The haplotype frequencies and the results of haplotype analyses with right VS activation magnitude and Binge Drinking Behavior frequency over the last 30 days. VS, ventral striatum*.

## Discussion

Using a two-stage translational approach, we first identified 42 genes whose expression level in prefrontal cortex across mouse strains correlated with impulsive behavior, and studied their association with human adolescent impulsivity. Variations in these genes were associated with an analogous form of human adolescent impulsivity and with activation of ventral striatum during reward anticipation, a potential contributory factor to impulsive behavior. The concordance between the mouse and human observations suggests that related biological events may underlie waiting impulsivity (premature responding) in the two species. Furthermore, we identified variations in a single gene, KALRN, which associated with levels of ventral striatal activation during reward anticipation, and with a propensity to engage in binge drinking in early adolescence.

We found significant strain differences in performance in the 5-CSRTT, a task now well-established for studying impulsivity in rodents (Robbins, 2002; see Sanchez-Roige et al., 2012 for a review). The parental B6 and D2 strains showed marked differences in impulsivity when tested under challenging conditions, though not during baseline performance. Differences between our findings which we have now replicated several times (Peña-Oliver et al., 2014; Sanchez-Roige et al., 2014b, 2015) and some previous findings (Pattij et al., 2007; Loos et al., 2010), may reflect differences in both procedure, and perhaps in substrains used (Sanchez-Roige et al., 2012).

In particular, the present findings were based on performance following the introduction of a long ITI session, whereas both previous reports (Pattij et al., 2007; Loos et al., 2010, 2014) employed data from the training phase of the task. An informal comparison of our data, and those of Loos et al. (2014), for those strains that were tested in both laboratories (*n* = 8) shows moderate negative correlation for the premature response data (Rho = −0.310, n.s., correlation performed in http://www.GeneNetwork.org). Examination of our own data reveals that the long ITI premature responses did not correlate significantly with premature responses during the training phase suggesting that these measures reflect different aspects of behavior. We have suggested elsewhere (Sanchez-Roige et al., 2012) that rodents may use two strategies during performance of the 5-CSRTT, internal timing to predict the timing of onset of the light stimulus, and the position of the stimulus to indicate the correct response location. Introduction of the long ITI condition would then disrupt the usefulness of internal timing, leading to premature responding. Thus, analyses employing training phase data may be more likely to include factors involved in timing behavior, while performance following introduction of the long ITI may provide a better measure of impulsivity (i.e., failure to withhold responses).

High levels of impulsivity are associated with several forms of drug-taking in rodents (Dalley et al., 2007; Belin et al., 2008; Diergaarde et al., 2008) and humans (DeVito et al., 2013; Stautz and Cooper, 2013). In the current experiments this association is replicated insofar as the B6 strain, which is consistently described as presenting high levels of ethanol drinking and high rates of self-administration of ethanol and cocaine (see Grahame and Cunningham, 1995; Crawley et al., 1997 for a review; Rocha et al., 1998), showed a high level of impulsivity in comparison with the low impulsive/low drinker D2 strain.

Correlational analyses of the mouse impulsivity data showed associations between expression levels of 42 genes in prefrontal cortex, and only one gene in accumbens and in striatum, using a false discovery rate of < 0.05 (Benjamini et al., 2001). Even though these correlations were carried out without accounting for population structure, subsequent analysis excluding parental strains (in order to lessen the issue of relatedness) also revealed a high correlation between kalirin and mouse impulsivity (rho = −0.8166, *p* = 0.004). Moreover, the finding from the human analysis linking kalirin and VS activation during reward anticipation, gives additional support to this correlational association between kalirin and the impulsive phenotype both in the mouse and human populations, although the causal relationship of this association will need to be addressed in future studies.

Importantly, when treated as a group, variations in the impulsivity-associated genes identified in the mouse were also statistically associated with premature responding in human adolescents, as well as with VS activation during performance of the MID task. As mentioned above, further analysis revealed that a single gene, *KALRN*, also associates with the VS activation measure. Although the MID is primarily a measure of reward anticipation, premature responding during the task may reflect an impaired ability to delay responding under conditions of high reward anticipation, and is thus analogous to rodent premature responding in 5-CSRTT. Interestingly, we found a moderate positive association between premature responding and VS activation. Although VS activation is conventionally interpreted as indicating the involvement of this area in reward anticipation, the present observations suggest that VS activation may also contribute to control over premature responding. That the mouse genetic findings mapped on to the human observations confirms the usefulness of investigations with recombinant inbred strains of mice as a strategy for identifying candidate genes for further analysis in human populations, providing the behavioral phenotypes under study in the two species are sufficiently homologous.

Impulsive behaviors may result from deficits in response inhibition, involving the top-down cognitive control exerted by the PFC in its interaction with striatal structures (Dalley et al., 2011), and projections from the anterior cingulate/ventromedial prefrontal cortex to the ventral striatum (and reciprocal interconnections) are known to mediate impulsivity (Robbins, 2007; Brewer and Potenza, 2008; Fineberg et al., 2010). Thus, altered connectivity in PFC (such as that arising from changes in kalirin function) is likely to be reflected in activity within the ventral striatum (VS). Consistent with that idea, fMRI BOLD-response in VS during reward anticipation correlated with percentage of premature responses in the MID task.

Furthermore, the G allele (major) of the intronic SNP rs6438839 in the KALRN gene was significantly associated with increased right VS activation and close to significance for the left VS activation. A second SNP, rs4634050, within the same KALRN haplotype block associated with “Binge drinking in the previous 30 days.” This association is of particular interest as a recent report indicates that children of alcoholics, who are themselves at risk for developing alcohol abuse, also show altered activation of these same areas during MID reward anticipation (Yau et al., 2012), while non-alcohol abusing individuals with a family history of alcoholism also show greater impulsivity in a human version of the 5-CSRTT (Sanchez-Roige et al., under review).

*KALRN* encodes kalirin, a guanine-nucleotide exchange factor that is expressed both within, and outside the CNS (Mandela et al., 2012). The gene is complex with multiple promoters and several 3′-untranslated regions which produce distinct isoforms that are functionally distinct and tissue specific. However, since the haplotype we identify is common to all isoforms (Figure 4B), it seems unlikely that these haplotypic variations contribute differentially to the formation of particular isoforms. The protein, too, is complex, with several catalytic domains interacting with both protein and lipid substrates (Mandela et al., 2012). In the CNS, kalirin is a key regulator of spine morphogenesis (Cahill et al., 2009), as well as dendritic outgrowth and branching (Yan et al., 2015). Kalirin is an essential component of mature excitatory synapses, interacting with multiple PDZ-domain-containing proteins including PSD95, spinophilin, and GluR1 through its PDZ-binding motif (Mandela and Ma, 2012).

In cultured hippocampal/cortical neurons, overexpression of Kalirin-7, the major isoform, increases spine density and spine size whereas reduction of endogenous Kalirin-7 expression decreases synapse number, and spine density. Deletion of Kalirin-7 results in reductions in spine length, synapse number, and postsynaptic density (PSD) in hippocampal pyramidal neurons, and these morphological alterations are accompanied by a deficiency in long-term potentiation (LTP) and a decreased spontaneous excitatory postsynaptic current (sEPSC) frequency (Mandela and Ma, 2012). Changes in spine size and number are associated with a number of physiological, behavioral, and pathological conditions, including drug addictions (Robinson and Kolb, 1997, 2004; Zhou et al., 2007). Importantly, circuit-level analysis indicates that relatively small changes in synapse strength or number may have a disproportionate effect on the overall function of circuits (Chklovskii et al., 2004; Chen and Nevidi, 2010; Mandela and Ma, 2012), so that even minor changes in kalirin function may have marked effects on circuit activity and behavior. Lentiviral knockdown of the kalirin-7 splice variant within accumbens of rats in adulthood leads to a reduction in cocaine self-administration in rats (Wang et al., 2013), while constitutive knockout of kalirin-7 in mice resulted in increased cocaine self-administration, especially at lower doses of cocaine (Kiraly et al., 2013). The basis of these seemingly contradictory findings may conceivably lie in developmental compensatory mechanisms in the constitutive knockout.

In humans, kalirin has been associated with a number of disorders in which impulsiveness is a feature (Remmers et al., 2014), including ADHD (Lesch et al., 2008), a condition characterized by lowered impulse control, that is also associated with altered frontal cortical control of striatal function (Robbins, 2007; Cubillo et al., 2012; Whelan et al., 2012). However, we did not find a significant association of *KALRN* SNP variations with premature responding in the MID, indicating that the association found between the polygenic score and premature responding is likely to be attributable to small contributions from a number of genes. Nevertheless, our findings are consistent with the efficiency of fronto-striatal connections depending upon kalirin-dependent spine formation either within prefrontal cortex, or potentially on striatal medium spiny neuron targets of glutamatergic outputs from cortex. These targets are also implicated in kalirin-dependent medium spiny neuron plasticity induced by cocaine (Mains et al., 2011).

Previous studies of impulsivity measured in the 5-CSRTT have emphasized the roles of dopamine and serotonin (Pattij and Vanderschuren, 2008; Kirby et al., 2011). Surprisingly, we found no evidence of associations of impulsivity with expression levels of genes related to monoamine neurotransmission. This failure to find an association may be attributable to the relatively low number of strains used in the present study, resulting in only limited power to detect such associations. However, it is also possible that changes in monoaminergic transmission nevertheless arise as a consequence of altered translation, posttranslational modification, or localization of receptors. In particular, monoaminergic inputs into ventral striatum serve to modulate the effectiveness of excitatory glutamatergic signals, and alterations in glutamatergic input would be expected to disrupt this modulation without necessarily depending upon altered expression of monoamine-related genes.

To conclude, our present results indicate the utility of the strategy of identifying candidate genes in mouse studies in complex, well-characterized behavioral tests that can then be used to increase the power of human genetic studies of homologous behaviors serving as behavioral endophenotypes for complex human disorders. Using this approach we identified one gene, *Kalrn*, to be associated with premature responding during reward anticipation in the mouse, while variations in the human homolog were associated with increased ventral striatum activation during reward anticipation and premature responding, suggesting a common underlying neural mechanism. Lastly, the association of a gene that is essential in synaptic spine formation with both impulse control and adolescent alcohol abuse provides a novel insight into the neurobiology of these conditions.

## Author contributions

All authors listed, have made substantial, direct and intellectual contribution to the work, and approved it for publication.

### Conflict of interest statement

The authors declare that the research was conducted in the absence of any commercial or financial relationships that could be construed as a potential conflict of interest.
